# Impact of Liver and Primary Tumor Localization on Survival in Lung Metastasectomy for Colorectal Carcinoma

**DOI:** 10.5761/atcs.oa.25-00056

**Published:** 2025-10-24

**Authors:** Salih Duman, Eren Erdoğdu, Arda Sarıgül, Şeyhmus Çuhatutar, Berker Özkan, Adalet Demir, Murat Kara

**Affiliations:** Department of Thoracic Surgery, Istanbul University Istanbul Medical Faculty, Istanbul, Turkey

**Keywords:** colorectal carcinoma, lung metastasectomy, thoracoscopy

## Abstract

**Purpose:**

Despite the knowledge that right-sided colon tumors are associated with worse overall survival (OS) and disease-free survival (DFS) compared to left-sided and rectal tumors, there are conflicting results on the impact of the primary location of colorectal tumors after lung metastasectomy. In this study, we aimed to investigate this contradiction.

**Methods:**

We conducted a retrospective analysis of 131 patients who underwent lung metastasectomy for colorectal cancer. DFS and OS were evaluated in relation to primary tumor location, liver metastasis, lymph node dissection, nodal status, and carcinoembryonic antigen (CEA) levels.

**Results:**

Among patients with no nodal metastasis (N0), significantly better DFS (p = 0.024) and OS (p = 0.030) were observed. Elevated CEA levels were not associated with DFS but were linked to improved OS (p = 0.004). Right-sided colon tumors were associated with worse DFS and OS compared to left-sided and rectal tumors (p <0.002 and p <0.001, respectively).

**Conclusion:**

Right-sided colon tumors were associated with the poorest DFS and OS, underscoring the prognostic significance of primary tumor location. Additionally, the absence of nodal metastasis was associated with significantly improved survival outcomes. Liver metastasis was not significantly associated with DFS or OS.

## Introduction

Colorectal cancer (CRC) ranks as the third most commonly diagnosed cancer and the third leading cause of cancer-related mortality among both men and women worldwide.^1)^ The lungs are the second most frequent site of metastasis in CRC, occurring in approximately 10%–20% of patients.^2)^ Right-sided and left-sided colorectal tumors exhibit distinct characteristics in multiple aspects. Right-sided tumors are more frequently diagnosed in older patients, tend to present at a more advanced stage, and have a higher prevalence of microsatellite instability and BRAF mutations. Furthermore, right-sided tumors are frequently linked to poorer differentiation and lower overall survival (OS) rates compared to left-sided tumors.^3,4)^ Wang et al., in a study using the Surveillance, Epidemiology, and End Results (SEER) database, reported that right-sided tumors in metastatic CRC were associated with worse survival outcomes.^5)^ However, other studies have reported conflicting findings, indicating that patients with CRC may exhibit similar disease-free survival (DFS) and OS regardless of tumor location.^6)^

Lung metastasectomy is a widely recognized surgical approach for patients with metastatic CRC, offering potential survival benefits for carefully selected cases. Despite advances in systemic therapies, surgery remains a cornerstone in the management of oligometastatic disease, providing both curative and palliative outcomes. The European Society For Medical Oncology Clinical Practice Guideline defines the requirements for patient selection as follows: the primary tumor must be radically resected, and all detected metastases must be treated by local treatments.^7)^

The presence of metastases other than in the liver or lung, older age (over 70), multiple pulmonary metastases, lung nodules larger than 2 cm, high carcinoembryonic antigen (CEA) levels before pulmonary resection, and mediastinal lymph node involvement have been previously identified as negative prognostic factors after pulmonary metastasectomy for metastatic CRC.^8,9)^ However, the impact of primary tumor location remains understudied, with conflicting results suggesting worse survival for rectal,^10)^ left-sided,^11)^ or right-sided tumors.^12)^

This study investigates the role of primary tumor location on survival outcomes in patients undergoing lung metastasectomy for CRC. By analyzing DFS and OS in relation to other clinical variables, we aim to provide insights that could guide clinical decision-making and improve long-term outcomes

## Materials and Methods

We retrospectively analyzed all the patients who underwent lung metastasectomy for metastatic CRC between June 2002 and June 2024 in our department. We included patients who underwent resection for CRC, and if present, the liver metastases were radically treated either by surgical resection, stereotactic body radiation therapy (SBRT), or radiofrequency (RF) ablation. All the metastasectomies were performed with curative intent. Patients with incomplete clinical or follow-up data were excluded. All patients were evaluated preoperatively by a multidisciplinary thoracic oncology board.

The indications for lung metastasectomy included complete resection of the primary tumor, absence of tumors in other organs, and complete resectability of lung metastases. In cases where patients had liver metastases, they underwent surgical resection, SBRT, or RF ablation.

Patients were selected based on the presence of histologically confirmed colorectal and resectable pulmonary metastases.

### Surgical procedures

Minimally invasive techniques, including video-assisted thoracic surgery (VATS) and robotic-assisted thoracic surgery (RATS), were used when feasible. All surgeries aimed to achieve the smallest resection possible while ensuring complete removal of metastatic lesions. Thoracotomy was chosen for patients with complex or multiple metastases requiring thorough exploration or palpation, as well as for those with significant pleural adhesions. Wedge resection was performed for peripheral tumors, and segmentectomy was preferred if the tumor was limited to a lung segment and centrally located. All resected lesions were confirmed as metastases using on-site frozen section analysis during surgery. For patients with bilateral metastases, sequential surgeries were performed, starting with the side that had the largest or greatest number of metastatic lesions, with the second surgery scheduled at least 3 weeks after discharge.

### Follow-up

Postoperative follow-up was conducted in collaboration with the oncology, colorectal surgery, and thoracic surgery teams. DFS was defined as the time from lung metastasectomy to the detection of new metastases in the lungs or other organs, or local recurrence in the colon.

### Data collection

Demographic, clinical, and pathological data, including age, sex, primary tumor location (right-sided colon, left-sided colon, rectum), presence of liver metastasis, lymph node status, CEA levels, type of lung surgery (wedge resection, lobectomy, segmentectomy), and the time spent from colorectal surgery to lung metastasectomy, were collected from patient medical records. Survival outcomes, including DFS and OS, were assessed.

### Statistical analysis

Data analysis was performed using Microsoft Excel (version 16.0; Microsoft Corporation, Redmond, WA, USA) and SPSS (Statistical Package for Social Sciences, version 25.0; IBM Corporation, Chicago, IL, USA). The Kolmogorov–Smirnov test was used to assess the normality of data distribution through histogram examination. Descriptive statistics, including mean, median, percentage, and standard deviation, were calculated, and continuous variables were compared using the Student’s t-test or the Mann–Whitney U-test.

Survival analyses were conducted using the Kaplan–Meier method to estimate OS and DFS rates. Survival differences between groups were evaluated using the log-rank test. The Cox proportional-hazards model was applied for regression analysis to determine the prognostic impact of the factors on survival. Statistical significance was defined as a p value <0.05.

## Results

We included 131 patients who underwent lung metastasectomy for primary colon cancer. The mean age of the patients was 57.2 ± 11.5 years (range: 20–79 years). The cohort consisted of 77 males (58.8%) and 54 females (41.2%). Mediastinal lymph node dissection was performed in 121 patients (92.4%). Among the patients who underwent lymph node dissection, 108 (81.7%) had no nodal metastasis (N0), 9 (6.9%) had N1, and 5 (3.9%) had N2 metastasis. Liver metastasis was present in 39 patients (29.1%). Regarding the primary tumor location, 61 patients (46.6%) had rectal cancer, 48 (36.6%) had left colon cancer, and 22 (16.8%) had right colon cancer. The metastatic lesions in the lung were located on the left side in 52 cases (39.7%), the right side in 51 cases (38.9%), and bilaterally in 28 cases (21.4%). Wedge resection was performed in 69 cases (52.7%). Anatomical resections were performed in 62 patients (47.3%), including lobectomies in 29 cases (22.1%) and segmentectomies in 33 cases (25.2%). The types of surgery included thoracotomy in 55 patients (42.0%), VATS in 73 patients (55.7%), sternotomy in two patients (1.5%), and RATS in one patient (0.8%). CEA levels had a mean value of 12 ± 45 (range: 0.74–361).

Patients with left-sided tumors were significantly older (mean age 60 ± 9.1 years) compared to those with right-sided (55 ± 12.3 years) and rectal tumors (55.4 ± 12.4 years, p = 0.041). Mediastinal lymph node dissection rates were similar between primary tumor locations, with 86.4% of right-sided, 93.8% of left-sided, and 93.4% of rectal tumors undergoing dissection (p = 0.508). The presence of lymph node metastasis also did not significantly differ among groups (p = 0.355). Liver metastases were slightly more common in patients with right-sided colon cancer (40.9%) compared to left-sided (35.4%) and rectal tumors (21.3%), but the difference was not statistically significant (p = 0.127). There was no significant difference in CEA levels between groups (p = 0.502). The distribution of lung metastases across left, right, and bilateral metastases was similar among tumor locations (p = 0.490). Bilateral lung metastases were more common in patients with right-sided colon cancer (27.3%) compared to left-sided (14.6%) and rectal tumors (24.6%) (p = 0.342). Regarding surgical approach, no significant differences were observed in the type of resection performed (wedge resection, lobectomy, segmentectomy; p = 0.531) or anatomical versus non-anatomical resections (p = 0.479). The rate of patients who underwent minimally invasive surgery was highest among rectal cancer patients (62.3%) compared to those with left-sided (50%) and right-sided tumors (63.6%), though the difference was not statistically significant (p = 0.366). The differences between the localization of the primary tumor and the clinicopathological characteristics are shown in **Table 1**. In the present analysis, comparing patients with right-sided colon tumors (n = 22) to those with tumors located in the left colon and rectum (n = 109), clinicopathological and surgical characteristics were examined to assess potential differences in metastatic lung resection outcomes among primary tumor locations. However, due to the absence of statistically significant differences in baseline or outcome parameters between these two groups, left-sided colon tumors and rectal tumors were subsequently combined into a single comparative group for the purposes of this analysis (**Supplementary Table 1**). After the combination statistics, demographic parameters, including age (55.0 ± 12.3 vs. 57.4 ± 11.3 years, p = 0.366) and sex distribution (male 59.1% vs. 58.7%, p = 0.948), were similar between the right-sided and left/rectal cohorts. Although some variation was observed in the distribution of colon cancer stages, this did not reach statistical significance (p = 0.077). A statistically significant difference was observed in the frequency of lymph node dissection, with fewer right-sided tumors undergoing nodal dissection compared to left/rectal tumors (86.4% vs. 93.6%, p = 0.028). Despite this, the incidence of lymph node metastasis did not differ significantly between groups (p = 0.257). Liver metastasis was more frequently observed in the left/rectal group (40.9% vs. 27.5%), although this difference did not achieve statistical significance (p = 0.127). Similarly, preoperative serum CEA levels were higher in the left/rectal group (15.3 ± 36 vs. 6.5 ± 10), without statistical significance (p = 0.502). Laterality of pulmonary metastases (unilateral vs. bilateral involvement) and the distribution of metastatic lesions across the left, right, and bilateral lung fields were comparable between the groups. While the extent of resection (e.g., wedge, segmentectomy, lobectomy) and the choice of surgical access (minimally invasive vs. open thoracotomy/sternotomy) did not differ significantly, a statistically significant difference was observed in the type of resection. Lobectomy was more frequently performed in patients with right-sided primary tumors (86.4% vs. 76.1%, p = 0.019), possibly reflecting a more extensive disease burden or anatomical considerations in this subgroup.

**Table 1:** Comparison of clinicopathological characteristics based on primary tumor location in metastatic colorectal cancer patients undergoing lung metastasectomy

	Right colon tumors, N (%)/mean ± SD	Left colon tumors, N (%)/mean ± SD	Rectum tumors, N (%)/mean ± SD	p Value
Number of patients	22 (16.8)	48 (36.6)	61 (46.6)	
Age	55 ± 12.3	60 ± 9.1	55.4 ± 12.4	**0.041**
Sex				
Male	13 (59.1)	28 (58.3)	36 (59)	
Female	9 (40.9)	20 (41.7)	25 (41)	0.997
Colon stage				
Stage I	3 (13.6)	2 (4.2)	5 (8.2)	
Stage IIA	4 (18.2)	15 (31.3)	17 (27.7)	
Stage IIB	9 (40.9)	11 (22.9)	13 (21.3)	
Stage IIIA	3 (13.6)	8 (16.7)	9 (14.8)	
Stage IIIB	3 (13.6)	12 (25)	17 (27.9)	0.553
Lymph node dissection				
Absent	3 (13.6)	3 (6.3)	4 (6.6)	
Present	19 (86.4)	45 (93.8)	57 (93.4)	0.508
Lymph node metastasis				
No nodal metastasis	2 (10.5)	3 (6.7)	9 (15.8)	
Nodal metastasis	17 (89.5)	42 (93.3)	48 (84.2)	0.355
Liver metastasis				
Absent	13 (59.1)	31 (64.6)	48 (78.7)	
Present	9 (40.9)	17 (35.4)	13 (21.3)	0.127
CEA	6.5 ±10	25 ± 81	7.6 ± 18	0.502
Lung side				
Left	6 (27.3)	21 (43.8)	25 (41.2)	
Right	10 (45.4)	20 (41.7)	21 (34.4)	
Bilateral	6 (27.3)	7 (14.5)	15 (24.6)	0.490
Side				
Unilateral	16 (72.7)	41 (85.4)	46 (75.4)	
Bilateral	6 (27.3)	7 (14.6)	15 (24.6)	0.342
Surgery				
Wedge	12 (54.5)	22 (45.8)	35 (57.3)	
Lobectomy	3 (13.7)	14 (29.2)	12 (19.7)	0.531
Segmentectomy	7 (31.8)	12 (25)	14 (23)	
Surgical type				
Anatomical resection	12 (54.5)	22 (45.8)	35 (57.4)	
Non-anatomical resection	10 (45.5)	26 (54.2)	26 (42.6)	0.479
Surgical decision				
Minimally invasive	14 (63.6)	24 (50)	38 (62.3)	
Thoracotomy–sternotomy	8 (36.4)	24 (50)	23 (37.7)	0.366

Statistically significant results are highlighted in bold.

CEA: carcinoembryonic antigen

**Fig. 1:**
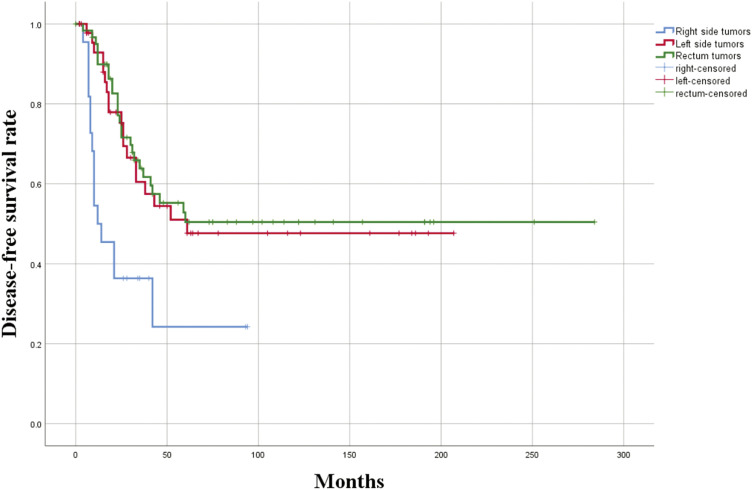
Disease-free survival according to the localization of the primary tumor localization.

**Fig. 2:**
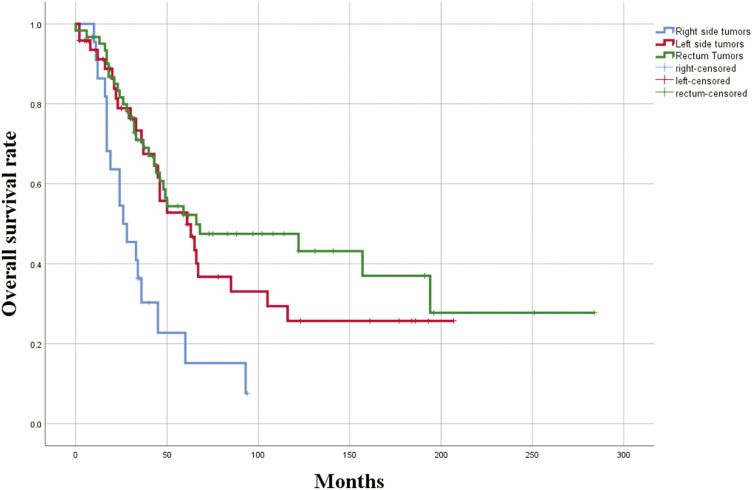
Overall survival according to the localization of the primary tumor localization.

No significant differences were observed in DFS or OS when analyzed by age, sex, side of lung surgery, bilateral versus unilateral surgery, type of surgery (wedge resection vs. lobectomy), anatomical versus non-anatomical resection, minimally invasive versus conventional surgery, or the time elapsed from colon surgery to metastasectomy. Patients who underwent lymph node dissection demonstrated significantly better outcomes in both DFS (49.1% vs. 20%, p = 0.045) and OS (64.9% vs. 20%, p = 0.080) compared to those who did not undergo lymph node dissection. Among patients who underwent lymph node dissection, the absence of nodal metastasis (N0) was associated with significantly better DFS (51.3% vs. 30.8%, p = 0.024) and OS (51.1% vs. 25.6%, p = 0.030) compared to those with nodal metastasis. CEA levels were not associated with DFS. However, OS was significantly higher in patients with elevated CEA levels (63.7% vs. 0%, p = 0.004). Patients with right-sided colon tumors had worse DFS (24.2%) compared to those with left-sided colon tumors (51%) and rectal tumors (50.4%; p = 0.002) (**Fig. 1**). Similarly, OS was significantly lower for right-sided colon tumors (15.2%) compared to left-sided colon tumors (52.8%) and rectal tumors (52.2%; p = 0.001) (**Fig. 2**). Clinicopathological features and their relationships with 5-year DFS and OS are shown in **Table 2**. The Kaplan–Meier also showed no statistically significant differences in survival between left-sided and rectal primary tumors in terms of DFS (p = 0.77) or OS (p = 0.39). Progression-free survival (PFS) was calculated from the time of primary colon tumor resection to the occurrence of lung metastasis. In the analysis, PFS was significantly associated with age (p = 0.048), mediastinal lymph node dissection (p = 0.004), and the primary tumor location within the colon (p = 0.006).

**Table 2:** Clinicopathological features and the 5-year DFS and OS of the cohort

	N (%)	PFS	p Value for PFS	DFS	p Value for DFS	OS	p Value for OS
Overall		66.9		64.7		46	
Age							
<58	59 (45)	67.6		33		45.5	
≥58	72 (55)	56.8	0.048	61.9	0.063	46.8	0.784
Sex							
Female	54 (41.2)	76.6		46.9		53	
Male	77 (58.8)	74.2		47.1	0.363	41.1	0.127
Colon stage							
Stage I	10 (7.6)	120		68.6		69.8	
Stage IIA	36 (27.5)	84.2		39.2		45.1	
Stage IIB	33 (25.2)	86.6		51.8		58.3	
Stage IIIA	20 (15.3)	107.1		74.5		80.7	
Stage IIIB	32 (24.4)	79.2	0.296	53.5	0.118	56.4	0.347
Lymph node dissection							
Absent	10 (7.6)	30		20		25	
Present	121 (92.4)	76.3	0.004	49.1	**0.045**	64.9	**0.080**
Lymph node metastasis							
No nodal metastasis	108 (81.7)	78.6		51.3		51.1	
Nodal metastasis	23 (18.3)	76.6	0.352	30.8	**0.024**	25.6	**0.030**
Liver metastasis							
Absent	92 (70.2)	76.7		52		50	
Present	39 (29.1)	63.1	0.108	35	0.079	35.3	0.198
CEA							
<14.5	43 (87.7)	81.7		63.2		63.7	
>14.5	6 (12.3)	66.7	0.388	62.5	0.721	0	**0.004**
Colon side							
Rectum	61 (46.6)	75.3		50.4		52.2	
Left colon	48 (36.6)	83		51		52.8	
Right colon	22 (16.8)	42.7	0.006	24.2	**0.002**	15.2	**0.001**
Lung side							
Left	52 (39.7)	75.6		50.8		42.3	
Right	51 (38.9)	68.3		41.5		46.1	
Bilateral	28 (21.4)	74.4	0.564	48.1	0.642	52.6	0.818
Side of metastasectomy							
Unilateral	103 (78.6)	71.9		46.1		44.3	
Bilateral	28 (21.4)	74.4	0.762	48.1	0.835	52.6	0.574
Surgical type							
Wedge	69 (52.7)	69.6		42.2		43.8	
Lobectomy	29 (22.1)	65.5		48.1		40.1	
Segmentectomy	33 (25.2)	83.4	0.510	54.1	0.613	56.5	0.509
Time to occurrence of lung metastasis							
≤36	74 (56.4)	57.8		48.9		45.7	
>36	55 (43.6)	89.1	0.071	44.1	0.919	48.9	0.349

Statistically significant results are highlighted in bold.

OS: overall survival; DFS: disease-free survival; CEA: carcinoembryonic antigen

Univariate analysis demonstrated that lymph node dissection, the presence of lymph node metastasis, and liver metastasis were not significantly associated with DFS or OS. However, the primary tumor location showed a significant impact on survival outcomes. Right-sided colon tumors were associated with significantly worse DFS (hazard ratio [HR] 2.806, 95% confidence interval [CI]: 1.552–5.069, p = 0.001) and OS (HR 2.718, 95% CI: 1.573–4.694, p <0.001) compared to left-sided colon and rectal tumors, as shown in **Table 3**.

**Table 3:** Univariate regression analysis of the cohort

	Disease-free survival			Overall survival		
	HR	CI	p Value	HR	CI	p Value
Lymph node dissection	2.101	0.995–4.438	0.079	1.846	0.916–3.719	0.076
Lymph node metastasis N2 + 1 versus N0	2.041	0.958–4.438	0.065	1.986	1.007–3.915	0.116
Liver metastasis	1.588	0.941–2679	0.083	1.370	0.844–2.222	0.298
Colon side (left and rectum vs. right colon)	2.806	1.552–5.069	**0.001**	2.718	1.573–4.694	**<0.001**

Statistically significant results are highlighted in bold.

HR: hazard ratio; CI: confidence interval

## Discussion

Our study aimed to assess the impact of primary tumor location and liver metastasis on survival outcomes in patients undergoing lung metastasectomy. Right-sided tumors showed the worst OS and DFS, and the highest impact on OS and DFS more important than mediastinal metastasis and liver metastasis.

The cohort displayed similar clinicopathological characteristics across different primary tumor locations. Factors that may impact survival, such as liver metastasis, mediastinal lymph node involvement, age, and bilateral lung metastases, were evenly distributed among the three groups. However, the rectal cancer group had a higher number of patients, aligning with previous literature. Patients with left-sided tumors were slightly older than those in the other groups, a factor that has been associated with poorer survival outcomes in cases of left-sided tumor metastasis.^9)^ Despite this, right-sided tumors continued to show the worst prognosis.

Meguid et al. conducted a SEER analysis on 77978 patients with both left- and right-sided colon adenocarcinoma and found that OS was significantly better with left-sided tumors. On the contrary, another SEER database analysis by Cai et al. reported better survival for right-sided neuroendocrine carcinoma of the colon in the presence of liver metastasis. They claimed that the survival dependence on the side of the primary colon tumor highly differs with the presence of liver metastasis and the histopathological subtypes. We believe our findings also highlight further aspects of prognostic features.^13,14)^

Several factors have been discussed in relation to different surgical outcomes of CRC. Right-sided tumors, which originate embryologically from the midgut, should be considered distinct entities from left-sided and rectal tumors, which develop from the hindgut. Right-sided tumors are characterized by a higher prevalence of microsatellite instability and exhibit 40% greater chromosomal instability than left-sided tumors.^15)^ Mutation rates in tumor suppressor genes and oncogenes also differ. Additionally, the mutation profiles differ, with right-sided tumors more frequently showing BRAF mutations, while left-sided tumors show higher rates of APC and KRAS mutations.^5)^ Moreover, responses to systemic therapy vary based on tumor location. As demonstrated by the PRIME study, adding panitumumab to FOLFOX improved survival for left-sided metastatic colorectal tumors but not for right-sided ones.^16)^ These studies suggest that survival outcomes are influenced not solely by the metastasectomy itself but also by the intrinsic biological characteristics of the primary tumor.

Our findings indicate that the presence of liver metastasis did not significantly affect DFS or OS in patients undergoing lung metastasectomy. This aligns with a previous study by Shishido et al., which suggested that, with appropriate patient selection, the presence of liver metastases does not preclude the potential benefits of pulmonary metastasectomy. They reported comparable survival outcomes in patients with and without liver metastases undergoing lung metastasectomy, emphasizing the importance of complete resection and careful patient selection. Lung and liver metastases that can be treated locally are associated with a lower recurrence risk compared to metastases in other sites, such as the peritoneum or brain.^17)^

The significance of mediastinal lymph node metastases in CRC has become increasingly evident. In our study, we demonstrated both the positive impact of mediastinal lymph node dissection and the negative effect of N2 metastases. However, when compared to primary tumor localization, no statistically significant results were observed in the univariate analysis. A meta-analysis by Gonzalez et al. found that the presence of mediastinal or hilar lymph node metastases was associated with worse OS, with multiple nodal metastases leading to even poorer outcomes.^8)^ However, there is still no consensus regarding the necessity, extent, or method of lymphadenectomy—whether it should involve full dissection or limited sampling. While lymphadenectomy plays a role in assessing prognosis and determining the need for adjuvant therapy, its direct impact on survival remains unclear.^18)^ The poorer survival observed in our study among patients who did not undergo mediastinal lymphadenectomy may not necessarily be due to the absence of the procedure itself, but rather to the increased risk of undetected mediastinal metastases when no dissection is performed.

In our study, elevated CEA levels were not significantly associated with DFS but were linked to improved OS. This paradoxical finding may be attributed to the complex interplay between tumor biology and host factors. Some studies suggest that elevated preoperative CEA levels may reflect a more aggressive tumor phenotype, while others propose that a decline in CEA levels post-resection could indicate a favorable response to treatment. Further research is warranted to clarify the prognostic value of CEA in this context.^19)^

Our study has several limitations, including its retrospective nature and the potential for selection bias inherent in surgical series. Additionally, the relatively small sample size may limit the generalizability of our findings. Despite these limitations, our results underscore the importance of considering primary tumor location in the prognostic assessment of patients undergoing lung metastasectomy for CRC.

In conclusion, while the presence of liver metastasis did not significantly impact survival outcomes, primary tumor location emerged as a critical determinant of prognosis in patients undergoing lung metastasectomy for CRC. These findings highlight the need for a nuanced approach to patient selection and underscore the potential value of integrating tumor sidedness into clinical decision-making processes.

## Supplementary Material

Supp. Table 1 Comparison of clinicopathological characteristics based on primary tumor location in metastatic colorectal cancer patients undergoing lung metastasectomy

## Declarations

### Data availability

The datasets generated and/or analyzed during the current study are available from the corresponding author upon reasonable request.

## References

[1] Sung H, Ferlay J, Siegel RL, et al. Global cancer statistics 2020: GLOBOCAN estimates of incidence and mortality worldwide for 36 cancers in 185 countries. CA Cancer J Clin 2021; 71: 209–49. 33538338 10.3322/caac.21660

[2] Van Cutsem E, Cervantes A, Adam R, et al. ESMO consensus guidelines for the management of patients with metastatic colorectal cancer. Ann Oncol 2016; 27: 1386–422. 27380959 10.1093/annonc/mdw235

[3] Yang C-Y, Yen M-H, Kiu K-T, et al. Outcomes of right-sided and left-sided colon cancer after curative resection. Sci Rep 2022; 12: 11323. 35790871 10.1038/s41598-022-15571-2 PMC9256690

[4] Brouwer NP, Van der Kruijssen DE, Hugen N, et al. The impact of primary tumor location in synchronous metastatic colorectal cancer: differences in metastatic sites and survival. Ann Surg Oncol 2020; 27: 1580–8. 31792717 10.1245/s10434-019-08100-5 PMC7138773

[5] Wang C, Wainberg ZA, Raldow A, et al. Differences in cancer-specific mortality of right-versus left-sided colon adenocarcinoma: a surveillance, epidemiology, and end results database analysis. JCO Clin Cancer Inform 2017; 1: 1–9. 10.1200/CCI.17.00099 30657397

[6] Ouchi A, Sadachi R, Hamaguchi T, et al. Prognostic relevance of primary tumor sidedness in early-stage colorectal cancer: an integrated analysis of 4 randomized controlled trials (JCOG2003A). Ann Surg 2024; 279: 283–9. 37551612 10.1097/SLA.0000000000006076

[7] Cervantes A, Adam R, Roselló S, et al. Metastatic colorectal cancer: ESMO Clinical Practice Guideline for diagnosis, treatment and follow-up*. Ann Oncol 2023; 34: 10–32. 36307056 10.1016/j.annonc.2022.10.003

[8] Gonzalez M, Gervaz P. Risk factors for survival after lung metastasectomy in colorectal cancer patients: systematic review and meta-analysis. Future Oncology 2015; 11(sup2): 31–3. 10.2217/fon.14.259 25662325

[9] Okumura T, Boku N, Hishida T, et al. Surgical outcome and prognostic stratification for pulmonary metastasis from colorectal cancer. Ann Thorac Surg 2017; 104: 979–87. 28577846 10.1016/j.athoracsur.2017.03.021

[10] Cho JH, Hamaji M, Allen MS, et al. The prognosis of pulmonary metastasectomy depends on the location of the primary colorectal cancer. Ann Thorac Surg 2014; 98: 1231–7. 25086943 10.1016/j.athoracsur.2014.05.023

[11] Ampollini L, Gnetti L, Goldoni M, et al. Pulmonary metastasectomy for colorectal cancer: analysis of prognostic factors affecting survival. J Thorac Dis 2017; 9(Suppl 12): S1282–90. 29119016 10.21037/jtd.2017.07.100 PMC5653500

[12] Corsini EM, Mitchell KG, Correa A, et al. Effect of primary colorectal cancer tumor location on survival after pulmonary metastasectomy. J Thorac Cardiovasc Surg 2021; 162: 296–305. 32713636 10.1016/j.jtcvs.2020.03.181

[13] Meguid RA, Slidell MB, Wolfgang CL, et al. Is there a difference in survival between right- versus left-sided colon cancers? Ann Surg Oncol 2008; 15: 2388–94. 18622647 10.1245/s10434-008-0015-y PMC3072702

[14] Cai W, Ge W, Zhang J, et al. Primary tumor location (right versus left side of the colon) and resection affect the survival of patients with liver metastases from colonic neuroendocrine carcinoma: a population-based study. Therap Adv Gastroenterol 2021; 14: 17562848211036453. 10.1177/17562848211036453 PMC8558787 34733354

[15] Kane MF, Loda M, Gaida GM, et al. Methylation of the hMLH1 promoter correlates with lack of expression of hMLH1 in sporadic colon tumors and mismatch repair-defective human tumor cell lines. Cancer Res 1997; 57: 808–11. 9041175

[16] Boeckx N, Toler A, de Beeck KO, et al. Primary tumor sidedness impacts on prognosis and treatment outcome: results from three randomized studies of panitumumab plus chemotherapy versus chemotherapy or chemotherapy plus bevacizumab in 1st and 2nd line RAS/BRAF WT mCRC. Ann Oncol 2016; 27: vi27.

[17] Shishido Y, Ishii M, Maeda T, et al. Survival outcomes of lung metastases from colorectal cancer treated with pulmonary metastasectomy or modern systemic chemotherapy: a single institution experience. J Cardiothorac Surg 2023; 18: 327. 37964370 10.1186/s13019-023-02434-8 PMC10647062

[18] van Dorp M, Bousema JE, Torensma B, et al. Pulmonary metastasectomy with lymphadenectomy for colorectal pulmonary metastases: A systematic review. Eur J Surg Oncol 2022; 48: 253–60. 34656390 10.1016/j.ejso.2021.09.020

[19] Kankanala VL, Zubair M, Mukkamalla SKR. Carcinoembryonic Antigen. In: StatPearls[Internet]. Treasure Island (FL): StatPearls Publishing; 2025 Jan-. Updated 2024 Dec 11. 35201700

