# Supplementary material for: Impact of Liver and Primary Tumor Localization on Survival in Lung Metastasectomy for Colorectal Carcinoma
Source: Ann Thorac Cardiovasc Surg. 2025 Oct 24;31(1):25-00056. doi: 10.5761/atcs.oa.25-00056 (PMC12554383; doi:10.5761/atcs.oa.25-00056)
Supplement: Supp. Table 1 [file atcs-31-1-25-00056-s001.pdf]

Supp. Table 1. Comparison of clinicopathological characteristics based on primary tumor location in metastatic colorectal cancer patients undergoing lung metastasectomy

	**Right Colon Tumors**	**Left Colon and Rectum Tumors**	**p**
	N(%)/Mean ± SD	N(%)/Mean ± SD	
**Number of patients**	22 (16.8)	109 (84.2)	
**Age**	55 ± 12.3	57.4 ± 11.3	0.366
**Sex**			
Male	13 (59.1)	64 (58.7)	
Female	9 (40.9)	45 (41.3)	0.948
**Colon Stage**			
Stage I	3 (13.6)	7 (6.4)	
Stage IIA	4 (18.2)	32 (29.4)	
Stage IIB	9 (40.9)	24 (22.0)	
Stage IIIA	3 (13.6)	17 (15.6)	
Stage IIIB	3 (13.6)	29 (26.6)	0.077
**Lymph Node Dissection**			
Absent	3 (13.6)	7 (6.4)	
Present	19 (86.4)	102 (93.6)	**0.028**
**Lymph Node Metastasis**			
No nodal metastasis	2 (10.5)	12 (11.0)	
Nodal Metastasis	17 (89.5)	90 (82.6)	0.257
**Liver metastasis**			
Absent	13 (59.1)	79 (72.5)	
Present	9 (40.9)	30 (27.5)	0.127
**CEA**	6.5 ±10	15.3 ± 36	0.502
**Lung Side**			
Left	6 (27.3)	46 (42.2)	
Right	10 (45.4)	41 (37.6)	
Bilateral	6 (27.3)	22 (20.2)	0.193
**Side**			
Unilateral	16 (72.7)	87 (79.8)	
Bilateral	6 (27.3)	22 (20.2)	0.178
**Surgery**			
Wedge	12 (54.5)	57 (52.3)	
Lobectomy	3 (13.7)	26 (23.9)	
Segmentectomy	7 (31.8)	26 (23.9))	0.243
**Surgery Type**			
Minimally Invasive	14 (63.6)	62 (56.9)	
Thoracotomy-sternotomy	8 (36.4)	47 (43.1)	0.332
